# Supplementary material for: Relationship between uncertainty in the oil and stock markets before and after the shale gas revolution: Evidence from the OVX, VIX, and VKOSPI volatility indices
Source: PLoS One. 2020 May 5;15(5):e0232508. doi: 10.1371/journal.pone.0232508 (PMC7199967; doi:10.1371/journal.pone.0232508)
Supplement: Attachment — Submitted filename: Response to Reviewers.pdf [file pone.0232508.s002.pdf]

Response to Reviewers' Comments on Manuscript ID PONE-D-19-34694 entitled "Relationship between uncertainty in the oil and stock markets before and after the shale gas revolution: Evidence from the OVX, VIX, and VKOSPI volatility indices"

In response to the reviewers' comments, we have revised the previously submitted version. We thank reviewers for the valuable comments and suggestions to improve the paper. Our detailed explanation on how to revise the manuscript is given below point by point starting with a bullet •.

Reviewer(s)' comments:

Reviewer 1

Comments

Comment 1. Aside from revealing the lead-lag relationship among the variables employed in this study, authors had better mention why this study is important and why this study are able to contribute to the existing studies in the abstract section of this paper.

• Thank you for your valuable comment. As requested, we mentioned the importance and contribution to the existing studies in the abstract. The following sentence are added:

– **Abstract section, Page 1**: "In light of crude oils essential role in the world economy, the relationship between crude oil prices and stock markets is of great interest to finance practitioners and researchers. Previous studies usually use crude oil and stock price data to examine this relationship. This study contributes to the literature by investigating the links between crude oil and stock markets in terms of volatility indices. ... These results have important implications for the analysis of portfolio risk management and for assisting energy policymakers and traders in making effective decisions and investments, respectively"

Comment 2. Authors mention that observing oil price volatility and taking actions in response to its expected changes are essential for managing risk would be important for the relevant literature. We argue that the above concern might be already taken into account in the relevant studies. As a result, we document that authors have to mention the main differences between your study and other

relevant studies in detail. In addition, how to manage the risks in terms of oil price fluctuated considerably should be illustrated in detail as well.

• Thank you for your valuable comment. As requested, we mentioned the main differences between our study and other relevant studies in detail. The following sentence are added to the Introduction, Section Discussion in terms of shale gas and risk management and Section Concluding remarks:

- **Introduction section, Page 3, lines 79 – 86**: "There are two aspects of this study that differ from previous studies. The first is our use of volatility indices to identify the relationship between crude oil and the stock market. Although previous empirical studies find causal relationships between oil prices and stock indices, research on the causality between implied volatility indices is scarce. To bridge this gap, we adopt the OVX, VIX, and VKOSPI to measure the implied volatility in oil prices, the S&P 500, and the KOSPI 200, respectively. Second, whereas previous studies focus mainly on the relationship between crude oil and the stock market, we focus on the change in that relationship over time."

- **Discussion in terms of shale gas and risk management section, Page 19 – 20, lines 472 – 485**: "The difference between the existing studies and this study is that we use volatility indices to identify the relationship between crude oil and the stock market. The volatility index represents a measure of the risk and it implies the market participants' expectations for the market. Therefore, we can see whether the expected risk predicted by each market participant affects the expectations of other market participants by looking at the changes in the relationship between the volatility indices. Our empirical results indicate that forecasts of risks to crude oil and risks to the stock market were mutually influenced prior to 2014, but recently the effects have been reduced. Furthermore, we explain the relationships between the U.S. and South Korea stock markets, and between crude oil and the South Korean stock market with respect to the volatility indices. The studies that examine the relationship between crude oil and U.S. stock markets are quoted in Section 2. There are also studies on the effect of changes in crude oil prices on the South Korean economy(Masih et al. [2011],Ran and Voon [2012],Wang et al. [2013]), but they use crude oil prices and the South Korean stock market index not volatility indices. Likewise, Jeon and Jang [2004] and Kim and Ryu [2015] examine the relationship between stock prices in the U.S. and South Korean stock markets, but they use stock indices."

- **Concluding remarks section, Page 21, lines 536 – 539**: "This study is noteworthy in that the influence of crude oil volatility on the U.S. and South Korean stock markets has decreased significantly. In addition, it is remarkable that the influence of the U.S. stock market on the South Korean stock market has increased."

Furthermore, we added a section(Section Discussion in terms of shale gas and risk management) to state how to manage the risks in terms of oil price fluctuated in detail. In the section, we illustrated a way to manage the risk of oil price fluctuation for a portfolio of oil and stocks, according to the relevant literature. We mentioned the calculation of optimal portfolio weights and hedge ratios as a future study. The following sentence are added:

- **Discussion in terms of shale gas and risk management section, Page 20, lines 487 − 493**: "We can manage the volatility risk arising from the crude oil price fluctuations by calculating optimal portfolio weights and hedge ratios. Assume that an investor attempts to hedge exposure to crude oil price fluctuation for a portfolio of oil and stocks. Then, the investor wants to minimize the risk of his/her oil-stock portfolio without reducing its expected returns. According to Kroner and Ng [1998], conditional volatilities can be used to construct optimal portfolio weights

$$w_t^{\mathrm{S0}} = \frac{h_t^{\mathrm{S}} - h_t^{\mathrm{S0}}}{h_t^{\mathrm{O}} - 2h_t^{\mathrm{SO}} + h_t^{\mathrm{S}}}, \quad \text{with} \quad w_t^{\mathrm{S0}} = \begin{cases} 0, & \text{if } w_t^{\mathrm{S0}} < 0 \\ w_t^{\mathrm{S0}}, & \text{if } 0 \geq w_t^{\mathrm{S0}} \leq 1 \\ 1, & \text{if } w_t^{\mathrm{S0}} > 1 \end{cases}$$

  where w_t^{S0} is the weight of oil in the crude oil and stock portfolio at time t and $h_t^{\mathrm{S}}, h_t^{\mathrm{O}}$, and h_t^{SO} are the conditional volatility of oil and stock and conditional covariance between oil and stock returns at time t, respectively. Therefore, when we calculate the conditional volatilities by using some models, such as the GARCH and BEKK models, we obtain a dynamic hedge ratio. Furthermore, given an OVX derivatives and stocks portfolio, we can also apply this process to calculate the optimal hedge ratio."
- **Concluding remarks section, Page 21, lines 558 − 559**: "Possible future studies include research on optimal portfolio weights and hedge ratios with respect to the sub-period data used in this study."

Comment 3. The motivation of this study should be enhanced and appeared in the front of this paper since readers prefer the strong motivation presented in the beginning of the introduction section.

• Thank you for your valuable comment. As requested, we enhanced the motivation of this study and presented it in the Introduction section. The following sentence are added:

- **Introduction section, Page 2, lines 42 − 51**: "Most research still uses crude oil and stock prices. However, the volatility indices are a better suitable barometer of the fragility of the markets and the economy. Therefore, the aim of this work is to investigate the relationship among the volatility indices, to derive important implications for the analysis of portfolio risk management. Furthermore, since the

introduction of volatility derivatives (e.g., Chicago Board Options Exchange (CBOE) volatility index (VIX) futures, options, and exchange-traded products), the trading volume has been increasing because they can be used as a risk-hedging strategy against stock market downturns (e.g. Park [2016]). Accordingly, investigation of the relationship between volatility indices can give necessary insight into suggestions for the pricing of volatility derivatives."

Comment 4. Authors had better provide the reasons why authors adopt these methodologies (i.e. the autoregressive distributed lag (ARDL) bounds test of cointegration as well as the TodaYamamoto (TY) Granger causality test of Toda and Yamamoto (1995)). Why not employing other methodologies due to one of the methodologies proposed in 1995? Are there any other update and appropriate methodologies likely employed for this paper?

• Thank you for your valuable comment. As requested, we introduced the cointegration test and mentioned the reason why we adopt the ARDL bounds test. About the ARDL bounds test, the following sentence are added:

– **ARDL bounds tests subsection, Page 9 – 10, lines 199 – 208**: "The cointegration tests proposed by Engle and Granger [1987], Johansen [1991], and Johansen and Juselius [1990] have been used in many empirical studies to investigate the long-run relationship of economic variables. However, the use of these approaches is limited. For example, these methods can be applied to those series that have a unique order of integration. The ARDL bounds test proposed by Pesaran and Shin [1998] and Pesaran et al. [2001] is a popular method because it has certain advantages over traditional cointegration methods.

First, it does not need all the variables in the model to be integrated of the same order. Second, the approach is relatively more efficient in the case of small and finite sample data sizes (Pesaran and Shin [1998] and Tang [2002]). Third, applying the ARDL technique, we obtain unbiased estimates of the long-term model (Harris and Sollis [2003])."

About TY Granger causality test, we introduced the causality test and mentioned the reason why we adopt the TY Granger causality test. Furthermore, we cited some studies in which TY causality test is used. We emphasize the following sentence:

– **TY Granger causality tests subsection, Page 11, lines 236 – 247**: "Granger [1969] proposes a test of the causal relationship between two variables, known as Granger causality. A time series (X) is said to Granger cause another time series (Y) if the prediction error of the current Y declines by using the past values of X in addition to the past values of Y. In the test, the two variables are expressed by

simple vector autoregression (VAR). The Granger causality test is easy to implement and can be applied in many types of empirical studies. Nonetheless, it also has some drawbacks. According to Shirazi and Manap [2005], first, the Granger causality test for inferring the leads and lags among integrated variables can provide spurious regression results. Second, it does not consider the effect of the number of lags even though this can affect the performance of a causality test. In other words, the results of the Granger causality test depend on the number of lags. Moreover, Toda and Phillips [1993] insist that Granger causality can lead to drawing wrong conclusions because of the dependence of the parameters."

– **TY Granger causality tests subsection, Page 11, lines 264 – 274**: "The TY Granger causality test has several advantages over other methods. First, it can provide a valid result regardless of whether a series is $I(0), I(1)$, or $I(2)$, not cointegrated, or cointegrated of any arbitrary order. Second, the TY test avoids the bias associated with unit root and cointegration tests (Zapata and Rambaldi [1997],Clarke and Mirza [2006]) as it does not require pre-testing of the cointegrating properties of the system. Third, we can explore the causality between variables with a possibly integrated and cointegrated system using the augmented VAR model in the TY test because the long-run information of the system in the general VAR model often disappears in the mandatory process of first differencing and pre-whitening (Clarke and Mirza [2006],Jain and Ghosh [2013]). Therefore, we adopt TY causality testing in this study. Furthermore, many recent studies adopt the approach of identifying causality using the TY causality test (Adriana [2014],Ghosh and Kanjilal [2014],Ziramba [2015],Faisal et al. [2017],Sankaran et al. [2019])."

In addition, we referred to developed methodologies for Causality test since TY Granger causality test of Toda and Yamamoto (1995)). They are nonlinear version, asymmetric, and bootstrap panel causality tests. We mentioned the nonlinear causality tests as a future study. The following sentence are added:

– **TY Granger causality tests subsection, Page 11, lines 254 – 263**: "Several other methodologies have been developed since the TY causality test was introduced. In view of the TY causality test being limited to finding linear cause-effect relationship, nonlinear version Granger causality tests have been developed(e.g. Hiemstra and Jones [1994] and Kyrtsou and Labys [2006]). We can detect nonlinear interactions between the variables using these tests. By contrast, Hatemi-j [2012] proposes an asymmetric causality test for the existence and direction of causality. Thus, the causality between positive and negative shocks of variables can be determined in the method. In addition, Kónya [2006] introduces a bootstrap panel causality method in order to account for both cross-sectional dependence and slope heterogeneity(Kar et al. [2011]). The approach is widely used to test for causality in a panel framework in many empirical studies."

– **Concluding remarks section, Page 21, lines 559 – 562**: "In addition, we can consider the causality between the positive and negative shocks of volatility indices by using nonlinear causality tests. In other words, it is necessary to ascertain how they affect and receive each other when market risk increases and when it decreases."

Comment 5. In fact, ARCH effects are likely existed in financial time series. We suggest that authors have better employ Multivariate GARCH-family models to examine the relationships among the variables (i.e. OVX, VIX, and VKOSPI) employed in this study for robustness.

• Thank you for your valuable comment. As requested, we employ BEKK-GARCH model as multivariate GARCH-family model to examine the relationships among volatility indices. We briefly introduced BEKK-GARCH model in subsection Multivariate GARCH model and added empirical results by the BEKK-GARCH model were given in subsection BEKK-GARCH(1,1) model and section Sub-period analysis. These results are also consistent with the causality test results obtained by TY Granger causality test.

Comment 6. The contribution of this study could be enhanced since the presentation of the contribution of this study might not appeal readers.

• Thank you for your valuable comment. As requested, we enhanced the contribution of this study and mentioned three main contributions in the Introduction. The following sentence are added:

– **Introduction section, Page 3, lines 87 – 103**: "We obtain three main contributions from these differences. The first is the investigation of the relationship between future expectations for each market—the crude oil, U.S., and South Korean stock markets. In particular, the volatility index represents the future risk measure of market participants. Therefore, we can investigate the relationship between the risk measures implied by crude oil, the S&P 500 index, and the KOSPI 200 index by using the volatility indices. The second is the examination of the causality between the OVX and VKOSPI and between the VIX and VKOSPI. To the best of our knowledge, this study is the first to investigate the relationship between the OVX and VKOSPI. Based on their relationship, policymakers can propose laws and policies for oil-importing countries to manage market risk. As mentioned above, South Korea and the United States have a close economic relationship; hence, it is reasonable to explore the causality between them owing to the uncertainty in their stock markets. The third major contribution concerns the change in the relationship between the volatility indices as revealed through a sub-period analysis. Based on the empirical results of the sub-period analysis, we conclude that one of the factors causing

the change in the relationship is the increased production of shale gas. Detailed discussions on this will be covered in Section 6."

In order to appeal readers, we presented some applications and implications based on this empirical results. The following paragraph is added to the Section Concluding remarks:

– **Concluding remarks section, Page 21, lines 540 – 557**: "We present some applications and implications based on this studys results. First, for both investors and policymakers, the key application of our work is properly forecasting financial market volatility. In particular, changes in the VIX in the U.S. stock market are strongly related to those in the South Korean stock market. In other words, we can increase the predictive power of the future VKOSPI in the South Korean stock market using the movement of the VIX. As Gong and Lin [2018] claim, forecasting volatility indices may be more beneficial to the decision-making of all stock market participants (including financial traders, manufacturers, and policymakers). Second, because volatility indices are used to hedge volatility risk, our findings will help to manage volatility risk in crude oil portfolios. According to Chen et al. [2011] and Liu et al. [2013], OVX derivatives, futures, and options can be a financial tool to hedge volatility risk. Furthermore, volatility indices have become a popular asset class for investors considering diversifying their portfolio strategy. Thus, our empirical findings can be used to examine and evaluate volatility derivatives, such as OVX options and futures. Third, contrary to expectations, South Korea, an emerging market, has not been sensitive to crude oil risks lately. There are many reasons for this, but oil import diversification may be one of them. Therefore, this study can be seen as evidence of the effect of crude oil import diversification for oil-importers, and in particular, South Korea."

Comment 7. Due to the data period employing in this study from January 2, 2009 to December 28, 2018, we argue that authors had better to examine whether there are any structural changes existed over the data period instead of examining the relationship among these variables by using two almost equal sub-periods (i.e. two sub-periods, namely January 2009May 2014 (sub-period 1) and June 2014December 2018 (sub-period 2).

• As requested, we examined the structural changes over the data period and calculated structural breakpoints for OVX time series by the algorithm described in [Bai and Perron, 2003]. In addition, we referred to some studies in which the sub-period analysis was investigated. The following sentence are added:

– **Sub-period analysis section, Page 16, lines 383 – 390**: "In addition to investigating the relationship between the volatility indices for the entire period (2009–2018), the total sample is examined for structural breaks in OVX by using the Bai and Perron [2003] sequential breakpoint tests. According to the breakpoint tests, the entire sample is split into two sub-periods after locating the date of 10/8/2014 as the breakpoint. Therefore, we analyze two sub-periods, namely January 2, 2009–October 7, 2014 (sub-period 1), and October 8, 2014–December 28, 2018 (sub-period 2). The OVX time series for the two sub-periods are illustrated in Fig 2 with the breakpoint. This sub-period analysis is often carried out in other studies(Nazlioglu et al. [2013], Guesmi and Fattoum [2014], Kayalar et al. [2017], Pavlova et al. [2018])."

FIGURE 2. **The sub-periods and breakpoint for the OVX time series.**

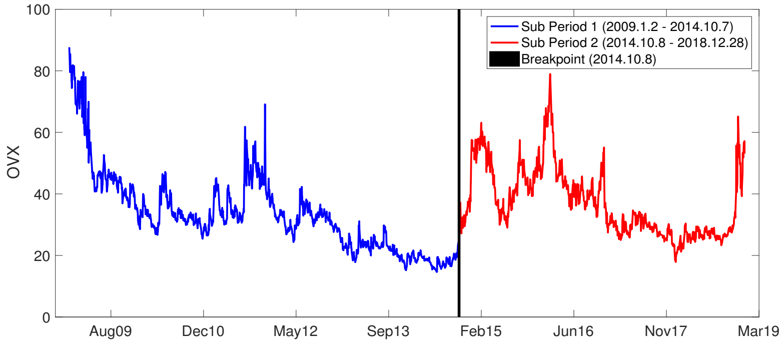

Reviewer 2

Summary of the contents and Comments
This paper studies the long-term relationships among the volatility indices of OVX, VIX, and VKOSPI while shale gas revolution happens and use the methodologies of ARDL and Toda-Yamamoto granger causality test. The results show that there is a bidirectional causality between OVX and VIX with the shale gas revolution and also finds some unidirectional causalities from VIX to VKOSPI. The topic is interesting but the current quality of the paper below the minimum acceptance level required/requested by the journal. I would suggest authors to improve the paper in accordance with the comments below.

Comment 1. First of all, this paper lacks solid theoretical backgrounds and motivations. Most of the journal readers with finance/economics backgrounds might understand that the oil and stock markets would affect with each other but, for other readers in different areas might need to learn more such relationships. A brief discussion as I mentioned above should be added in the paper. After that, authors should further develop the relationships among these volatility indices.

• Thank you for your valuable comment. As requested, we added a brief discussion about the relationship between oil and stock markets with several studies in the Introduction. The following sentence are added:

– **Introduction section, Page 1-2, lines 7 – 14**: "Oil affects industrial development significantly and oil prices have naturally been the subject of global attention over the past several decades. A rise in crude oil prices increases the production cost of the manufacturing industry, reducing corporate profitability, which has a negative effect on stock prices (e.g. Barsky and Kilian [2004]). This is because increased crude oil price volatility can negatively affect economic growth, causing greater economic uncertainty. Empirical test results indicating that crude oil prices and economic activity are very much related are already seen in many studies(Papapetrou [2001],Brown and Yücel [2002],Cunado and De Gracia [2005],Lescaroux and Mignon [2008],He et al. [2010],Jo [2014],Herrera et al. [2015],Kayalar et al. [2017], Boubaker and Raza [2017],Chiang et al. [2019], Dong et al. [2019])."

Comment 2. Authors should add a section of Literature Review to comprehensively review the relationships among these volatility indices.

• As requested, we added the section of Literature Review and we presented a review of the literature on volatility indices in Section Literature Review.

Comment 3. In the current version, I did not see the discussion of shale gas revolutions and have no idea how authors define this revolution. In addition, authors should provide more explanations why the sample period is divided into two sub-periods.

• Thank you for this comment. First, we mentioned the sub-period analysis in the Introduction. In addition, we explained why we implement the sub-period analysis in detail and we examined the structural changes over the data period in Section 5. Sub-period analysis. We cited a few relevant studies in which the sample period is divided into two sub-periods to support our approach. The structural breakpoints for OVX time series are calculated by the algorithm described in [Bai and Perron, 2003]. The following paragraph is added to the Introduction and Section 5:

– **Introduction section, Page 3, lines 72 – 78**: "Furthermore, these tests are explored using a sub-period analysis to examine whether their relationship is constant over time, which would provide insight into the dynamic nature of the interactions between the volatility indices. The other reason we proceed with the sub-period analysis is because we want to analyze how shale gas, an alternative to crude oil,

affects the relationship between stock markets and the oil market. Several studies investigate the effect of the shale gas revolution on the oil market."

– **Sub-period analysis section, Page 16, lines 383 – 390**: "In addition to investigating the relationship between the volatility indices for the entire period (2009–2018), the total sample is examined for structural breaks in OVX by using the Bai and Perron [2003] sequential breakpoint tests. According to the breakpoint tests, the entire sample is split into two sub-periods after locating the date of 10/8/2014 as the breakpoint. Therefore, we analyze two sub-periods, namely January 2, 2009–October 7, 2014 (sub-period 1), and October 8, 2014–December 28, 2018 (sub-period 2). The OVX time series for the two sub-periods are illustrated in Fig 2 with the breakpoint. This sub-period analysis is often carried out in other studies(Nazlioglu et al. [2013], Guesmi and Fattoum [2014], Kayalar et al. [2017], Pavlova et al. [2018])."

As requested, we added a section(Section Discussion in terms of shale gas and risk management) dealing with the problem to intensively discuss it. In the section, we displayed the the annual shale gas production, the exports and imports of crude oil data obtained from U.S. Energy Information Administration and we suggested that shale gas production has affected the import and export of crude oil based on the data after 2014. According to Kilian [2016], the price of oil experienced one of its largest declines in modern history between June 2014 and December 2014. Therefore, we define the shale gas revolution as a sharp rise in shale gas production to the extent that it can affect the amount of oil export and imports. Based on the sub-sample periods, the following sentence are added:

– **Discussion in terms of shale gas and risk management section, Page 19, lines 429 – 434**: "In the previous section, we use the two sub-periods, January 2, 2009–October 7, 2014 (sub-period 1), and October 8, 2014–December 28, 2018 (sub-period 2). According to Kilian [2016], the price of oil experienced one of its largest declines in modern history between June 2014 and December 2014. Therefore, we can regard these two sub-periods as the period during which the shale gas revolution took place (sub-period 2) and the period before it happened (sub-period 1), respectively."

Lastly, we discussed the sub-period analysis results in terms of shale gas revolution. Our conclusion is that shale gas is the one of main factors that has caused the change of the relationship between the two oil and stock markets. We cited some studies investigating the changes according to the shale gas production through sub-period analysis. The following sentence are added:

– **Discussion in terms of shale gas and risk management section, Page 19, lines 443 – 457**: "Of course, the shale gas revolution is not the only direct cause of the change in the relationship between the OVX and VIX. There are many factors that have affected the relationship between the two markets, but what we want to

argue here is that shale gas is the one of main factors that has caused the change. As you can see from Fig 3, the shale gas revolution would have had a major effect on the crude oil market and on the oil-related market and we regard the effect as a change in the relationship between the OVX and VIX. The arguments made through this research process can be found in other papers. To study the changes according to the shale gas production, there are several studies that have implemented sub-period analysis. Geng et al. [2016] examines the effect of the shale gas revolution on North American and European natural gas markets through two sub-periods, the pre-revolution period and the post-revolution period. Similarly, Li et al. [2017] investigate the effects of oil price shocks on the stock returns in the oil industrial chain companies and inspect the differences between the two periods. Although Li et al. [2017] focus on the oil price shock itself, they explain the shale gas solution as the cause of the oil price shock. ”

FIGURE 3. **U.S. shale production and export and import from 2007 to 2018.**

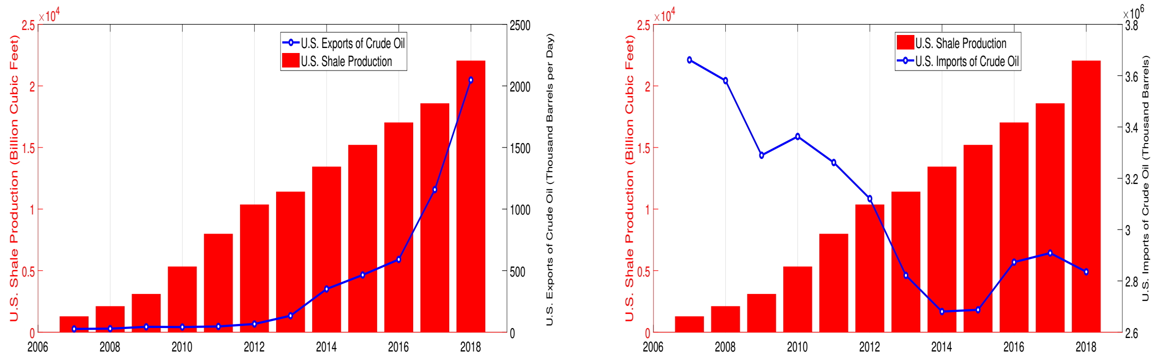

(a) Shale production and exports of crude oil

(b) Shale production and imports of crude oil

Comment 4. Several research might be included in the paper as follows:
Giot, P. (2005). Relationships between implied volatility indexes and stock index returns. The Journal of Portfolio Management, 31(3), 92-100.
Chiang, S. M., Chen, C. D., & Huang, C. M. (2019). Analyzing the impacts of foreign exchange and oil price on biofuel commodity futures. Journal of International Money and Finance, 96, 37-48.

• As requested, we have included many the relevant previous studies in this work, including the mentioned papers. The following sentence are added:

– **Literature Review section, Page 4, lines 116 – 117**: "For example, Giot [2005] investigates the relationship between the implied volatility and underlying stock index for both the S&P 100 and Nasdaq 100 indices."

– **Introduction section, Page 2, lines 12 – 14**: "Empirical test results indicating that crude oil prices and economic activity are very much related are already seen in many studies(Papapetrou [2001],Brown and Yücel [2002],Cunado and De Gracia [2005],Lescaroux and Mignon [2008],He et al. [2010],Jo [2014],Herrera et al. [2015],Kayalar et al. [2017], Boubaker and Raza [2017],Chiang et al. [2019], Dong et al. [2019])."

Comment 5. I would also like to see more financial/economic applications and implications based on the results found in the paper.

• Thank you for your comment. In the last Section, we mentioned a number of ways in which we could utilize the results found in this study. The following paragraph is added to the Section 7. Concluding remarks:

– **Concluding remarks section, Page 21, lines 540 – 557** "We present some applications and implications based on this studys results. First, for both investors and policymakers, the key application of our work is properly forecasting financial market volatility. In particular, changes in the VIX in the U.S. stock market are strongly related to those in the South Korean stock market. In other words, we can increase the predictive power of the future VKOSPI in the South Korean stock market using the movement of the VIX. As Gong and Lin [2018] claim, forecasting volatility indices may be more beneficial to the decision-making of all stock market participants (including financial traders, manufacturers, and policymakers). Second, because volatility indices are used to hedge volatility risk, our findings will help to manage volatility risk in crude oil portfolios. According to Chen et al. [2011] and Liu et al. [2013], OVX derivatives, futures, and options can be a financial tool to hedge volatility risk. Furthermore, volatility indices have become a popular asset class for investors considering diversifying their portfolio strategy. Thus, our empirical findings can be used to examine and evaluate volatility derivatives, such as OVX options and futures. Third, contrary to expectations, South Korea, an emerging market, has not been sensitive to crude oil risks lately. There are many reasons for this, but oil import diversification may be one of them. Therefore, this study can be seen as evidence of the effect of crude oil import diversification for oil-importers, and in particular, South Korea."

References

Davidescu Adriana. Revisiting the relationship between unemployment rates and shadow economy. a toda-yamamoto approach for the case of romania. *Procedia Economics and Finance*, 10:227–236, 2014.

Jushan Bai and Pierre Perron. Computation and analysis of multiple structural change models. *Journal of applied econometrics*, 18(1):1–22, 2003.

Robert B Barsky and Lutz Kilian. Oil and the macroeconomy since the 1970s. *Journal of Economic Perspectives*, 18(4):115–134, 2004.

Heni Boubaker and Syed Ali Raza. A wavelet analysis of mean and volatility spillovers between oil and brics stock markets. *Energy Economics*, 64:105–117, 2017.

Stephen PA Brown and Mine K Yücel. Energy prices and aggregate economic activity: an interpretative survey. *The Quarterly Review of Economics and Finance*, 42(2):193–208, 2002.

Hsuan-Chi Chen, San-Lin Chung, and Keng-Yu Ho. The diversification effects of volatility-related assets. *Journal of Banking & Finance*, 35(5):1179–1189, 2011.

Shu-Mei Chiang, Chun-Da Chen, and Chien-Ming Huang. Analyzing the impacts of foreign exchange and oil price on biofuel commodity futures. *Journal of International Money and Finance*, 96:37–48, 2019.

Judith A Clarke and Sadaf Mirza. A comparison of some common methods for detecting granger noncausality. *Journal of Statistical Computation and Simulation*, 76(3):207–231, 2006.

Juncal Cunado and F Perez De Gracia. Oil prices, economic activity and inflation: evidence for some asian countries. *The Quarterly Review of Economics and Finance*, 45(1):65–83, 2005.

Minyi Dong, Chun-Ping Chang, Qiang Gong, and Yin Chu. Revisiting global economic activity and crude oil prices: A wavelet analysis. *Economic Modelling*, 78:134–149, 2019.

Robert F Engle and Clive WJ Granger. Co-integration and error correction: representation, estimation, and testing. *Econometrica: journal of the Econometric Society*, pages 251–276, 1987.

Faisal Faisal, Turgut Tursoy, and Ozlem Ercantan. The relationship between energy consumption and economic growth: Evidence from non-granger causality test. *Procedia computer science*, 120:671–675, 2017.

Jiang-Bo Geng, Qiang Ji, and Ying Fan. How regional natural gas markets have reacted to oil price shocks before and since the shale gas revolution: A multi-scale perspective. *Journal of Natural Gas Science and Engineering*, 36:734–746, 2016.

Sajal Ghosh and Kakali Kanjilal. Oil price shocks on indian economy: evidence from toda yamamoto and markov regime-switching var. *Macroeconomics and Finance in Emerging Market Economies*, 7(1):122–139, 2014.

Pierre Giot. Relationships between implied volatility indexes and stock index returns. *The Journal of Portfolio Management*, 31(3):92–100, 2005.

Xu Gong and Boqiang Lin. The incremental information content of investor fear gauge for volatility forecasting in the crude oil futures market. *Energy Economics*, 74:370–386, 2018.

Clive WJ Granger. Investigating causal relations by econometric models and cross-spectral methods. *Econometrica: Journal of the Econometric Society*, pages 424–438, 1969.

Khaled Guesmi and Salma Fattoum. Return and volatility transmission between oil prices and oil-exporting and oil-importing countries. *Economic Modelling*, 38:305–310, 2014.

Richard Harris and Robert Sollis. *Applied time series modelling and forecasting*. Wiley, 2003.

Abdulnasser Hatemi-j. Asymmetric causality tests with an application. *Empirical Economics*, 43(1):447–456, 2012.

Yanan He, Shouyang Wang, and Kin Keung Lai. Global economic activity and crude oil prices: A cointegration analysis. *Energy Economics*, 32(4):868–876, 2010.

Ana María Herrera, Latika Gupta Lagalo, and Tatsuma Wada. Asymmetries in the response of economic activity to oil price increases and decreases? *Journal of International Money and Finance*, 50:108–133, 2015.

Craig Hiemstra and Jonathan D Jones. Testing for linear and nonlinear granger causality in the stock price-volume relation. *The Journal of Finance*, 49(5):1639–1664, 1994.

Anshul Jain and Sajal Ghosh. Dynamics of global oil prices, exchange rate and precious metal prices in india. *Resources Policy*, 38(1):88–93, 2013.

Bang Nam Jeon and Beom-Sik Jang. The linkage between the us and korean stock markets: the case of nasdaq, kosdaq, and the semiconductor stocks. *Research in international Business and Finance*, 18(3):319–340, 2004.

Soojin Jo. The effects of oil price uncertainty on global real economic activity. *Journal of Money, Credit and Banking*, 46(6):1113–1135, 2014.

Søren Johansen. Estimation and hypothesis testing of cointegration vectors in gaussian vector autoregressive models. *Econometrica: journal of the Econometric Society*, pages 1551–1580, 1991.

Søren Johansen and Katarina Juselius. Maximum likelihood estimation and inference on cointegrationwith applications to the demand for money. *Oxford Bulletin of Economics and statistics*, 52(2):169–210, 1990.

Muhsin Kar, Şaban Nazlıoğlu, and Hüseyin Ağır. Financial development and economic growth nexus in the mena countries: Bootstrap panel granger causality analysis. *Economic modelling*, 28(1-2):685–693, 2011.

Derya Ezgi Kayalar, C Coşkun Küçüközmen, and A Sevtap Selcuk-Kestel. The impact of crude oil prices on financial market indicators: copula approach. *Energy Economics*, 61: 162–173, 2017.

Lutz Kilian. The impact of the shale oil revolution on us oil and gasoline prices. *Review of Environmental Economics and Policy*, 10(2):185–205, 2016.

Jun Sik Kim and Doojin Ryu. Return and volatility spillovers and cojump behavior between the us and korean stock markets. *Emerging Markets Finance and Trade*, 51(sup1):S3–S17, 2015.

László Kónya. Exports and growth: Granger causality analysis on oecd countries with a panel data approach. *Economic Modelling*, 23(6):978–992, 2006.

Kenneth F Kroner and Victor K Ng. Modeling asymmetric comovements of asset returns. *The review of financial studies*, 11(4):817–844, 1998.

Catherine Kyrtsou and Walter C Labys. Evidence for chaotic dependence between us inflation and commodity prices. *Journal of Macroeconomics*, 28(1):256–266, 2006.

François Lescaroux and Valérie Mignon. On the influence of oil prices on economic activity and other macroeconomic and financial variables. *OPEC Energy Review*, 32(4):343–380, 2008.

Qiming Li, Ke Cheng, and Xiaoguang Yang. Response pattern of stock returns to international oil price shocks: From the perspective of chinas oil industrial chain. *Applied Energy*, 185:1821–1831, 2017.

Ming-Lei Liu, Qiang Ji, and Ying Fan. How does oil market uncertainty interact with other markets? an empirical analysis of implied volatility index. *Energy*, 55:860–868, 2013.

Rumi Masih, Sanjay Peters, and Lurion De Mello. Oil price volatility and stock price fluctuations in an emerging market: evidence from south korea. *Energy Economics*, 33(5): 975–986, 2011.

Saban Nazlioglu, Cumhur Erdem, and Ugur Soytas. Volatility spillover between oil and agricultural commodity markets. *Energy Economics*, 36:658–665, 2013.

Evangelia Papapetrou. Oil price shocks, stock market, economic activity and employment in greece. *Energy economics*, 23(5):511–532, 2001.

Yang-Ho Park. The effects of asymmetric volatility and jumps on the pricing of vix derivatives. *Journal of Econometrics*, 192(1):313–328, 2016.

Ivelina Pavlova, Maria E de Boyrie, and Ali M Parhizgari. A dynamic spillover analysis of crude oil effects on the sovereign credit risk of exporting countries. *The Quarterly Review of Economics and Finance*, 68:10–22, 2018.

M Hashem Pesaran and Yongcheol Shin. An autoregressive distributed-lag modelling approach to cointegration analysis. *Econometric Society Monographs*, 31:371–413, 1998.

M Hashem Pesaran, Yongcheol Shin, and Richard J Smith. Bounds testing approaches to the analysis of level relationships. *Journal of applied econometrics*, 16(3):289–326, 2001.

Jimmy Ran and Jan P Voon. Does oil price shock affect small open economies? evidence from hong kong, singapore, south korea and taiwan. *Applied Economics Letters*, 19(16): 1599–1602, 2012.

A Sankaran, Sanjay Kumar, K Arjun, and Mousumi Das. Estimating the causal relationship between electricity consumption and industrial output: Ardl bounds and toda-yamamoto approaches for ten late industrialized countries. *Heliyon*, 5(6):e01904, 2019.

Nasim Shah Shirazi and Turkhan Ali Abdul Manap. Export-led growth hypothesis: Further econometric evidence from south asia. *The Developing Economies*, 43(4):472–488, 2005.

Tuck Cheong Tang. Demand for m3 and expenditure components in malaysia: assessment from bounds testing approach. *Applied Economics Letters*, 9(11):721–725, 2002.

Hiro Y Toda and Peter CB Phillips. Vector autoregressions and causality. *Econometrica: Journal of the Econometric Society*, pages 1367–1393, 1993.

Yudong Wang, Chongfeng Wu, and Li Yang. Oil price shocks and stock market activities: Evidence from oil-importing and oil-exporting countries. *Journal of Comparative Economics*, 41(4):1220–1239, 2013.

Hector O Zapata and Alicia N Rambaldi. Monte carlo evidence on cointegration and causation. *Oxford Bulletin of Economics and statistics*, 59(2):285–298, 1997.

E Ziramba. Causal dynamics between oil consumption and economic growth in south africa. *Energy Sources, Part B: Economics, Planning, and Policy*, 10(3):250–256, 2015.
