# Supplementary material for: Relationship between uncertainty in the oil and stock markets before and after the shale gas revolution: Evidence from the OVX, VIX, and VKOSPI volatility indices
Source: PLoS One. 2020 May 5;15(5):e0232508. doi: 10.1371/journal.pone.0232508 (PMC7199967; doi:10.1371/journal.pone.0232508)
Supplement: Attachment — Submitted filename: Response to Reviewers.pdf [file pone.0232508.s006.pdf]

Response to Reviewers' Comments on Manuscript ID PONE-D-19-34694 entitled "Relationship between uncertainty in the oil and stock markets before and after the shale gas revolution: Evidence from the OVX, VIX, and VKOSPI volatility indices"

We thank you for your time and consideration on our submission (PONE-D-19-34694). We address the Reviewers comments and list of changes that we made to our manuscript according to their reports. The original Reviewers comments are provided in bold fonts. Our answers are described in a point-to-point manner starting with a bullet •. The appropriate changes made in the revised manuscript are highlighted.

Reviewer(s)' comments:

Reviewer 1

Comments

Comment 1. "This study contributes to the literature by investigating the links between crude oil and stock markets in terms of volatility indices."
The above contribution might not appeal to readers. We argue that authors should mention something new and something important related to this study, which might be able to persuade readers that this study would significantly contribute to the existing literature.

• Thank you for your valuable comment. As requested, we mentioned the importance and contribution to the existing studies in the abstract. The abstract has been modified as follows:

– **Abstract**: "In light of crude oils essential role in the world economy, the relationship between crude oil prices and stock markets is of great interest to finance practitioners and researchers. Previous studies usually use crude oil and stock price data to examine this relationship. Financial markets generally view volatility index as a measure of fear in the markets and economy. Therefore, we investigate the relationship among the volatility indices of crude oil and stock in order to derive important managerial implications. Furthermore, we evaluate the change in the relationship between the volatility indices through a sub-period analysis. These are the contributions of this study. Specifically, we examine the causal relationships among the crude oil, S&P 500 index, and KOSPI 200 index volatilities, adopting the autoregressive distributed

lag (ARDL) bounds test to determine the long-term relationship. Based on the cointegration results of the ARDL bounds test, we adopt the Toda–Yamamoto Granger causality test to identify the causal relationship between the variables. Furthermore, we employ a BEKK-GARCH model for enhancing the robustness of the causality test results. We also confirm the change in this relationship over time through a sub-period analysis, using periods that include and exclude the shale gas revolution. These experiments indicate that the OVX and VIX show bi-directional causality in the period that includes the shale gas revolution and no causality in the period that does not. Further, the OVX Granger causes the VKOSPI in the former period, but there is no causality between them in the latter period. Finally, we find strong unidirectional causality from the VIX to the VKOSPI in both sub-periods. The results support evidence of time-varying dependence among the crude oil, U.S., and South Korean markets. These results have important implications for the analysis of portfolio risk management and for assisting energy policymakers and traders in making effective decisions and investments, respectively."

Comment 2. While authors respond to these comments, readers would prefer that the adding context or revised context would be shown right below the response to comments, since readers might not prefer the presentation as shown below.
In addition, the same concerns for responding to other comments

• Thank you for your valuable comment. As requested, we put the adding context below the response to the comment.
