# Supplementary material for: Relationship between uncertainty in the oil and stock markets before and after the shale gas revolution: Evidence from the OVX, VIX, and VKOSPI volatility indices
Source: PLoS One. 2020 May 5;15(5):e0232508. doi: 10.1371/journal.pone.0232508 (PMC7199967; doi:10.1371/journal.pone.0232508)
Supplement: Attachment — Submitted filename: PLOS_One_Comments.docx [file pone.0232508.s001.docx]

**Comments**

1. Aside from revealing the lead-lag relationship among the variables employed in this study, authors had better mention why this study is important and why this study are able to contribute to the existing studies in the abstract section of this paper.
2. Authors mention that “observing oil price volatility and taking actions in response to its expected changes are essential for managing risk” would be important for the relevant literature. We argue that the above concern might be already taken into account in the relevant studies. As a result, we document that authors have to mention the main differences between your study and other relevant studies in detail. In addition, how to manage the risks in terms of oil price fluctuated considerably should be illustrated in detail as well.
3. The motivation of this study should be enhanced and appeared in the front of this paper since readers prefer the strong motivation presented in the beginning of the introduction section.
4. Authors had better provide the reasons why authors adopt these methodologies (i.e. the autoregressive distributed lag (ARDL) bounds test of cointegration as well as the Toda–Yamamoto (TY) Granger causality test of Toda and Yamamoto (1995)). Why not employing other methodologies due to one of the methodologies proposed in 1995? Are there any other update and appropriate methodologies likely employed for this paper?
5. In fact, ARCH effects are likely existed in financial time series. We suggest that authors have better employ Multivariate GARCH-family models to examine the relationships among the variables (i.e. OVX, VIX, and VKOSPI) employed in this study for robustness.
6. The contribution of this study could be enhanced since the presentation of the contribution of this study might not appeal readers.
7. Due to the data period employing in this study from January 2, 2009 to December 28, 2018, we argue that authors had better to examine whether there are any structural changes existed over the data period instead of examining the relationship among these variables by using two almost equal sub-periods (i.e. two sub-periods, namely January 2009–May 2014 (sub-period 1) and June 2014–December 2018 (sub-period 2).

Decision: Major revision
