# Supplementary material for: Relationship between uncertainty in the oil and stock markets before and after the shale gas revolution: Evidence from the OVX, VIX, and VKOSPI volatility indices
Source: PLoS One. 2020 May 5;15(5):e0232508. doi: 10.1371/journal.pone.0232508 (PMC7199967; doi:10.1371/journal.pone.0232508)
Supplement: Attachment — Submitted filename: PLOS_One_Comments20200326.docx [file pone.0232508.s005.docx]

**Comments:**

1. This study contributes to the literature by investigating the links between crude oil and stock markets in terms of volatility indices.”

The above contribution might not appeal to readers. We argue that authors should mention something new and something important related to this study, which might be able to persuade readers that this study would significantly contribute to the existing literature.

2. While authors respond to these comments, readers would prefer that the adding context or revised context would be shown right below the response to comments, since readers might not prefer the presentation as shown below.

The following sentences are added:

Discussion in terms of shale gas and risk management section, Page 20, lines 487 – 493:

Concluding remarks section, Page 21, lines 558 – 559:

In addition, the same concerns for responding to other comments

Decision; Major revision
