# Supplementary material for: Relationship between uncertainty in the oil and stock markets before and after the shale gas revolution: Evidence from the OVX, VIX, and VKOSPI volatility indices
Source: PLoS One. 2020 May 5;15(5):e0232508. doi: 10.1371/journal.pone.0232508 (PMC7199967; doi:10.1371/journal.pone.0232508)
Supplement: Attachment — Submitted filename: PLOS_One_Comments20200416A.docx [file pone.0232508.s007.docx]

**Comments:**

1. In fact, the abstract should be neat and focused on your main concern in this paper, so we suggest that the abstract should be condensed in accordance to the above-mentioned concern.

Decision: minor revision
